# Transition from Deep Regional Blocks toward Deep Nerve Hydrodissection in the Upper Body and Torso: Method Description and Results from a Retrospective Chart Review of the Analgesic Effect of 5% Dextrose Water as the Primary Hydrodissection Injectate to Enhance Safety

**DOI:** 10.1155/2017/7920438

**Published:** 2017-10-01

**Authors:** Stanley K. H. Lam, Kenneth Dean Reeves, An-Lin Cheng

**Affiliations:** ^1^Department of Family Medicine, Faculty of Medicine, The Chinese University of Hong Kong, Sha Tin, Hong Kong; ^2^KH Lam Musculoskeletal Pain Management and Sports Injury Centre, Kowloon, Hong Kong; ^3^The Hong Kong Institute of Musculoskeletal Medicine, Tsuen Wan, Hong Kong; ^4^Private Practice PM&R and Pain Management, Roeland Park, KS, USA; ^5^Department of Biomedical and Health Informatics, University of Missouri-Kansas City School of Medicine, Kansas City, MO, USA

## Abstract

Deep nerve hydrodissection uses fluid injection under pressure to purposely separate nerves from areas of suspected fascial compression, which are increasingly viewed as potential perpetuating factors in recalcitrant neuropathic pain/complex regional pain. The usage of 5% dextrose water (D5W) as a primary injectate for hydrodissection, with or without low dose anesthetic, could limit anesthetic-related toxicity. An analgesic effect of 5% dextrose water (D5W) upon perineural injection in patients with chronic neuropathic pain has recently been described. Here we describe ultrasound-guided methods for hydrodissection of deep nerve structures in the upper torso, including the stellate ganglion, brachial plexus, cervical nerve roots, and paravertebral spaces. We retrospectively reviewed the outcomes of 100 hydrodissection treatments in 26 consecutive cases with a neuropathic pain duration of 16 ± 12.2 months and the mean Numeric Pain Rating Scale (NPRS) 0–10 pain level of 8.3 ± 1.3. The mean percentage of analgesia during each treatment session involving D5W injection without anesthetic was 88.1%  ±  9.8%. The pretreatment Numeric Pain Rating Scale score of 8.3 ± 1.3 improved to 1.9 ± 0.9 at 2 months after the last treatment. Patients received 3.8 ± 2.6 treatments over 9.7 ± 7.8 months from the first treatment to the 2-month posttreatment follow-up. Pain improvement exceeded 50% in all cases and 75% in half. Our results confirm the analgesic effect of D5W injection and suggest that hydrodissection using D5W provides cumulative pain reduction.

## 1. Introduction

Deep regional blocks have been used for years to provide perioperative anesthesia for surgery and postoperative pain control [1]. In the management of patients with chronic pain, such as those with complex regional pain syndrome (CRPS) and postherpetic neuralgia, deep regional blocks, for example, stellate ganglion blocks (SGBs), serve as an alternative to other medical treatments. The mechanism of action of deep regional blocks or repeated peripheral focal nerve blocks for neuropathic pain remains unclear [2]. A benefit from repeated depolarization by a local anesthetic was originally proposed; however, the effects of this method with regard to the normalization of nerve physiology have not been confirmed [2]. More recently, the concept has emerged that fascial compression of nerves can occur in multiple locations and that part of the benefit of deep regional blocks may be through partial amelioration of fascial compression [3]. Nerve hydrodissection is a technique involving the use of fluid injection under pressure to purposely and more completely separate nerves from their surrounding tissue [4]. Ultrasound is used to guide the needles and fluid (hydro) is used to separate and release (dissect) the nerves from the surrounding soft tissue/fascia.

Potential safety concerns with any perineural injection method using an anesthetic include temporary muscular weakness and loss of protective sensation [5]. The rate of inadvertent intraneural injection under ultrasound guidance approximates 16%-17% [6, 7], although long-term sequelae appear to be quite rare [6, 7]. Furthermore, inadvertent intravascular injection may occur because of the frequent close proximity of nerves and vessels. Injection of a high volume of anesthetic for hydrodissection is associated with an increased risk of both dose-related systemic anesthetic toxicity and inadvertent intravascular injection. The use of 5% dextrose water (D5W) as the primary injectate for perineural injection during hydrodissection in the presence of chronic pain, particularly neuropathic pain, is receiving increasing attention [8–12]. D5W is also considered for use as a coadministration injectate along with noxious agents such as chemotherapeutics [13, 14] and microbiospheres [15] to decrease pain, as well as a means to separate nerves from fascia while decreasing the risk of anesthetic toxicity [16]. An independent-of-anesthetic analgesic potential of D5W has been demonstrated in a recent randomized controlled trial of epidural D5W injection versus saline injection for patients with back pain accompanied by either buttock or leg pain [17], with potential long-term efficacy suggested by long-term follow-up data in those patients [18].

## 2. Objectives

Although low-level studies have demonstrated the effectiveness of nerve hydrodissection, no high-level studies have been reported [4]. Performance of high-level studies will be facilitated by procedural methods that are reproducibly performed, consistent in clinical effect and safe. The objectives of this study were to illustrate reproducible methods of hydrodissection for deep nerve structures in the upper torso, including the stellate ganglion, brachial plexus, cervical nerve roots, and paravertebral spaces and gather preliminary data related to the analgesic effect and efficacy of D5W without lidocaine as the primary injectate during hydrodissection for patients with chronic neuropathic pain.

## 3. Materials and Methods

A formal letter of exemption allowing retrospective chart review was obtained from the International Cellular Medicine Society Institutional Review Board (ICMS-IRB). We reviewed consecutive outpatient charts for patients who underwent hydrodissection of the stellate ganglion, brachial plexus, cervical nerve roots, or paravertebral spaces for the management of pain with neuropathic characteristics. Videos and still photographs of these patients were all deidentified for use. Charts were consecutively reviewed to identify participants who received hydrodissection exclusively with D5W, with the use of lidocaine only for the placement of skin blebs. Chart selection continued until data from 100 treatments was available for analysis. Methods of hydrodissection utilized for these consecutively recruited patients were illustrated with the use of both anatomical diagrams and ultrasound images.

Neuropathic pain, for the purpose of this write-up, was defined in standard fashion as pain arising as a direct consequence of a lesion or disease affecting the somatosensory system either at the peripheral or at central level [19, 20]. Neuropathic pain is commonly characterized by allodynia, hyperalgesia, and/or changes in temperature sensation (e.g., burning or cold pain); the extent of the pain does not typically correspond to the extent of the nervous structure damage.

Ultrasound findings were not useful for the diagnosis of neuropathic pain unless the cervical nerve roots and brachial plexus were scanned. In case of unilateral lesions, a comparison of the cross-sectional area and echotextures of cervical nerve roots or the brachial plexus on the painful side with those on the contralateral side without pain generally showed that the painful side was larger in cross-sectional area [21].

The decision to hydrodissect was based on the following factors.

(1) A clinical diagnosis indicating that a neurogenic pain source is likely and knowledge of the corresponding involved deep nerve structures in patients with neuropathic pain, for example, dermatomes of the nerves involved in patients with postherpetic neuralgia

(2) Awareness of the effects of compression on the function of peripheral nerves

(3) Knowledge of all potential sites of compression of peripheral nerves, for example, the radial nerve at entry to and exit from the radial tunnel

(4) Experience regarding the appearance of peripheral nerves when they are encased in fascia, obtained by observing the “plumping up” of nerves upon freeing them from the surrounding fascial encasement

(5) Confidence to proceed with a higher volume of perineural injection in the absence of a risk of lidocaine toxicity, considering the lidocaine component is absent or negligible

Hydrodissection involved consistent fluid injection at all times during needle advancement, ostensibly to push away any small nerve fibers and avoid pain during advancement. This eliminated the need for anesthetic injection. Because fluid always leads needle advancement during hydrodissection and pushes away nerve structures, vessels, and other soft tissues, this technique, if performed properly, prevents soft tissue damage by the needle. Without inclusion of a local anesthetic, typical signs of motor blockade, such as Horner's syndrome, were not expected during stellate ganglion infiltration. Accordingly, the primary endpoint was pain reduction. Typically, 20–30 ml of fluid was utilized for each area of hydrodissection.

Adequacy of a particular hydrodissection procedure was based on patient symptoms, because visualization of fluid surrounding the nerve is only directly observable during hydrodissection of the brachial plexus and cervical nerve roots. In our experience, the analgesic effect of dextrose occurs within 5 min after deep regional hydrodissection for a variety of chronic neuropathic pain conditions. Accordingly, 5 min after completion of the initial procedure, the pain was rated on a 0–10 Numeric Pain Rating Scale (NPRS) using the question “how much pain do you have?” A score of 0 represented “no pain” and a score of 10 represented the “most severe pain imaginable.” If the pain was rated as 3/10 or less, the reported score was considered to represent the postprocedural pain level. If residual pain was rated as 4/10 or more, another regional procedure that was reasonably expected to affect the region of pain was performed, and pain was rated again at 5 min after the procedure. This process was repeated for up to three pertinent procedures, and the final pain level was that following the last hydrodissection procedure. The same sequence was performed during follow-up visits.

The primary measure for a potential intraprocedural analgesic effect of D5W hydrodissection was the mean difference between pretreatment and immediate (5 min) posttreatment NPRS scores.

Routine follow-up procedure in the primary investigator's office was to contact patients at 2 months after treatment to inquire about any further need for treatment and verbally obtain a final NPRS score to monitor the treatment efficacy. Data were analyzed using PASW 18 (Predictive Analytics 180 Software 18.0.0, IBM Corporation, 1 New Orchard Road, Armonk, New York 10504-1722). Descriptive statistics (means ± standard deviations) were reported at baseline and at each time point for NPRS scores.

The cumulative improvement in pain levels over time was determined by calculating the mean difference between pretreatment NPRS scores and those obtained at 2 months after the last treatment visit. The proportion of patients who achieved more than 50% and more than 75% pain reduction was calculated.

### 3.1. Description of Hydrodissection Procedures by Area

#### 3.1.1. Stellate Ganglion Hydrodissection

**Figure 1:**
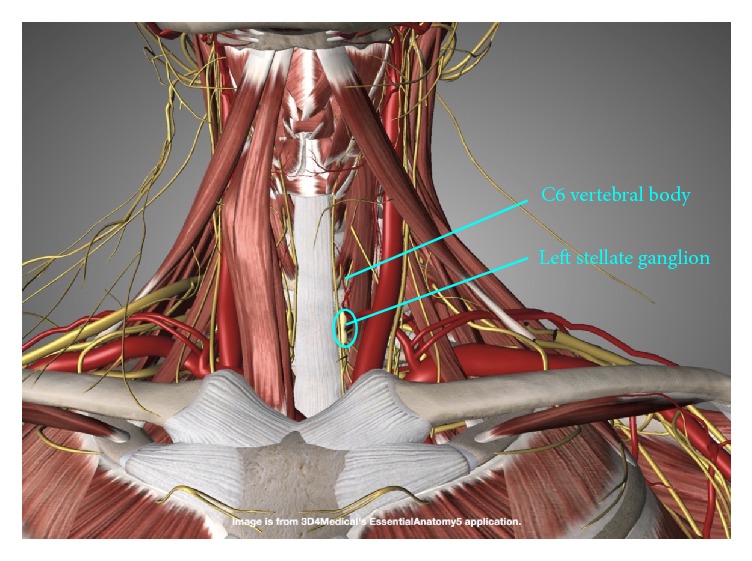
Longitudinal location of the stellate ganglion. The stellate ganglion is located at the level of the C7 vertebral body.

*Applications*. The stellate ganglion is part of the sympathetic network formed by the inferior cervical and first thoracic ganglia. It lies anterolateral to the C7 vertebral body (Figure 1), receives input from the paravertebral sympathetic chain, and provides sympathetic efferents to the upper extremities, head, neck, and heart. During pain management for CRPSs, particularly type I reflex sympathetic dystrophy (RSD) [22, 23], postherpetic neuralgia [24, 25], and chronic pain of the head and neck [26, 27] or thorax, a local anesthetic solution is injected as a local block for the stellate ganglion. Posttraumatic stress disorder [28, 29] may also be seen in these patients [30, 31]. Accordingly, its presence or absence was recorded, although the symptomatology was not assessed in the present study. Other applications of SGBs, such as vascular insufficiency and hyperhidrosis, were not within the scope of this study.

*Nonhydrodissection Methods*. A consecutive patient study described the efficacy and safety of using ultrasound to guide needles for SGB without the use of a high-volume technique [32]. Ultrasound guidance helps in the visualization of soft tissues to prevent complications and the subfascial deposition of the drug under direct vision [32, 33].

**Figure 2:**
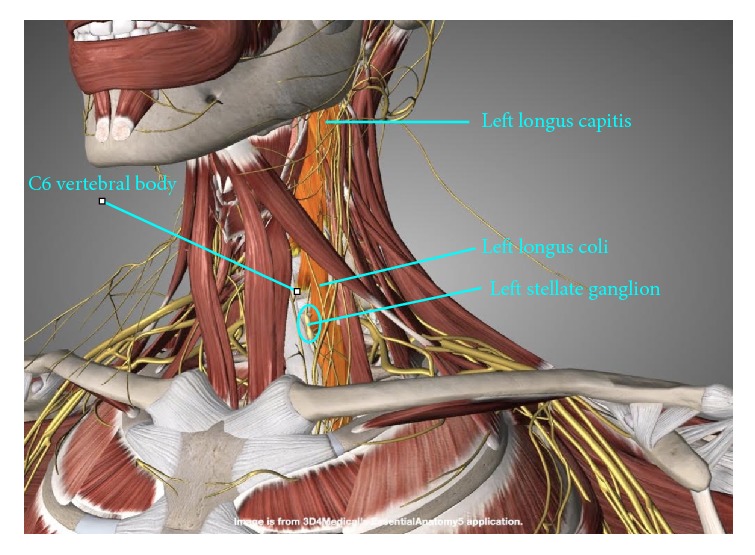
Relationship of the stellate ganglion to the longus colli. The stellate ganglion lies anterolateral to the C7 vertebral body and in the prevertebral fascia on the surface of the longus colli.

*Primary Ultrasound Landmarks for Hydrodissection*. The anterior tubercle of the C6 vertebral body, known as the Chassaignac tubercle or carotid tubercle, is an important landmark located superior to the stellate ganglion. C7 does not have an anterior tubercle, while the anterior tubercle of C5 is less prominent. Therefore, the anterior tubercle of C6 can be easily found. Identification of the longus colli is also key (Figure 2), as a cadaveric study using dye and clinical validation has shown adequate spread of the anesthetic solution to the stellate ganglion using a technique in which the needle tip is deep to the prevertebral fascia to avoid spread along the carotid sheath and superficial to the fascia investing the longus colli to avoid injection into the muscle substance [34].

**Figure 3:**
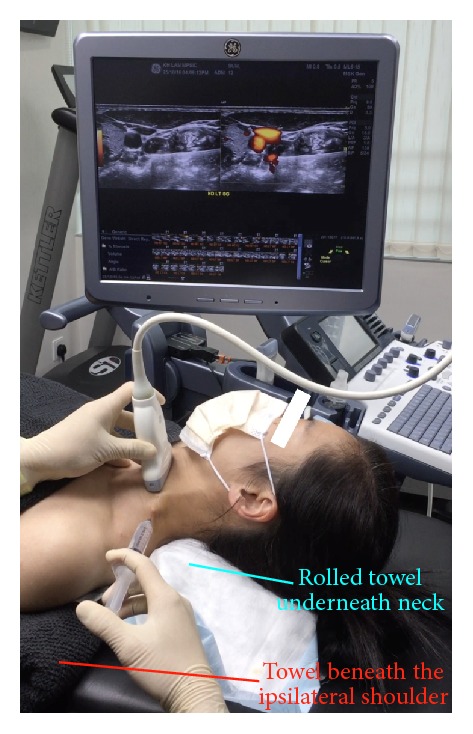
Procedure for stellate ganglion hydrodissection: patient positioning. Neck support, ipsilateral shoulder elevation, and probe and needle positions for stellate ganglion hydrodissection.

*Patient Position*. The patient is supine, with a rolled towel underneath the neck for slight extension and another thin pillow or rolled towel beneath the ipsilateral shoulder for slight rotation of the head to the side contralateral to the point of needle entry (Figure 3).

*Probe Position*. Probe placement is transverse to the neck. A lateral to medial in-plane approach is used, with the needle orientation slightly posterior to anterior (Figure 3).

**Figure 4:**
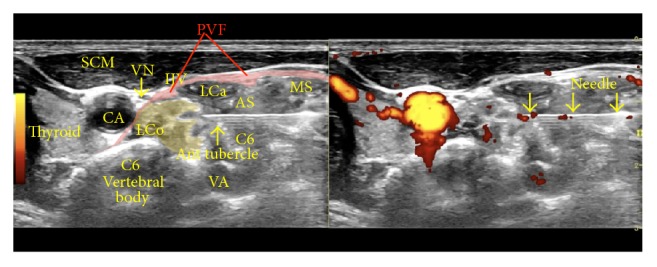
Procedure for stellate ganglion hydrodissection: sonoanatomy. The figure shows labeled structures, with the needle just past the anterior tubercle of C6. AS: anterior scalene, CA: carotid artery, IJV: internal jugular vein, LCa: Longus Capitis, LCo: longus colli, MS: middle scalene, PVF: prevertebral fascia, SCM: sternocleidomastoid, VN: vagus nerve, and VA: vertebral artery.

*Sonoanatomy*.  Figure 4 shows a dual sonographic image (left, B-mode; right, power Doppler) depicting the sonoanatomy and a sonographic view of the needle just past the anterior tubercle of the C6 vertebra (superior to the C6 nerve root and inferior to the C5 nerve root).

**Figure 5:**
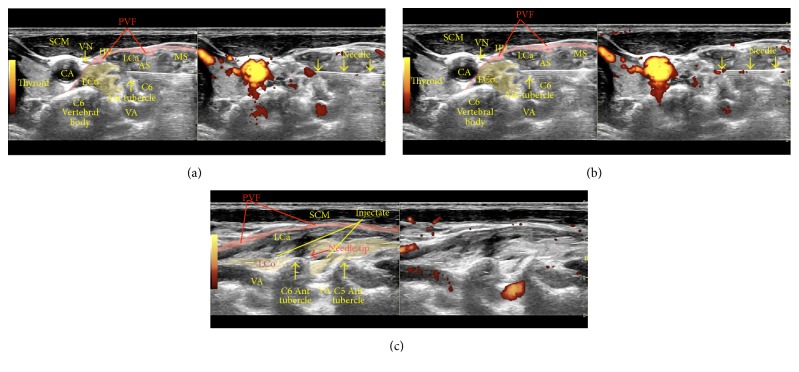
(a) Procedure for stellate ganglion hydrodissection: nearing the tip of the C6 anterior tubercle. Once the longus colli, anterior tubercle of C6, and the C6 nerve root are identified, advance the needle, with the bevel downwards, just ventral to the tip of the anterior tubercle of C6. AS: anterior scalene, CA: carotid artery, IJV: internal jugular vein, LCa: Longus Capitis, LCo: longus colli, MS: middle scalene, PVF: prevertebral fascia, SCM: sternocleidomastoid, VN: vagus nerve, and VA: vertebral artery. (b) Procedure for stellate ganglion hydrodissection: in the prevertebral fascia, superficial to the longus colli. Continue injecting (hydrodissecting) while advancing the needle to allow the fluid to open a channel for the needle when it is approaching in a direction from lateral to medial and posterior to anterior and passing through the middle scalene and in the space between the C5 and C6 nerve roots to approach the prevertebral fascia superficial to the longus colli. Stop advancing when the needle tip reaches the fascia, usually just anterior to the anterior tubercle of C6. (c) Procedure for stellate ganglion hydrodissection: observation of fluid tracking using sagittal images. The image shows the probe turned 90° to observe a sagittal image through the needle tip, which is next to the anterior tubercle of C6 at the insertion of the longus colli, for observation of fluid tracking in the prevertebral fascia superficial to the longus colli. The fluid will track down anterolaterally to the C7 vertebral body and reach the stellate ganglion.

*Needle Advancement/Injection*. Visualize the hypoechoic nerve roots situated between the anterior and posterior tubercles of the transverse processes of the cervical vertebrae. Locate the longus colli and the anterior tubercle of C6 and the C6 nerve root. It is essential to follow the basic principle of hydrodissection; that is, the fluid opens the channel or space in front of the needle tip, and the needle just follows. Advance the needle (Figure 5(a)) and stop advancing when the tip reaches the prevertebral fascia superficial to the longus colli (Figure 5(b)). After reaching the prevertebral fascia, turn the bevel of the needle down so that the injectate will push down the soft tissues in front of and beneath the needle. The idea is to use the force of the injectate to open a potential space between the prevertebral fascia and the longus colli. The tracking of the fluid beneath the prevertebral fascial can be further observed by turning the probe 90 degrees to show a sagittal image. Upon continued hydrodissection the fluid will be seen tracking caudally to reach the stellate ganglion (Figure 5(c)). Video 1 (in Supplementary Material available online at https://doi.org/10.1155/2017/7920438) shows the procedure for stellate ganglion hydrodissection.

*Treatment Frequency*. The effects may last from one to a few weeks depending on the severity of the symptoms. Typically, after 3–6 repeated treatments at 4–6-week intervals, the patient's pain will be relieved to a satisfactory level.

#### 3.1.2. Brachial Plexus Hydrodissection

*Applications*. Brachial plexus block is used for regional anesthesia during upper extremity surgery (arm, elbow, forearm, wrist, and hand) [35]. In chronic pain management, ultrasound-guided hydrodissection of the brachial plexus has been used to treat severe neck sprains (brachial plexus injury without rupture; it is only used to treat neuropraxia with or without axonotmesis) with radiating pain to the ipsilateral upper limb [36], CRPS [37] involving the ipsilateral upper limb, and thoracic outlet syndrome or other double/triple crush syndromes involving the ipsilateral upper limb [38].

*General Approaches and Selection of the Right Approach*. There are four different approaches/sites to perform brachial plexus hydrodissection: interscalene, supraclavicular [39], infraclavicular, and axillary [40]. Each approach has its own unique advantages and indications. Interscalene blocks are the most effective for anesthesia of the shoulder and proximal upper limb, while supraclavicular blocks are best suited for anesthesia from the mid-humerus to the fingers. Infraclavicular blocks are useful for procedures requiring continuous anesthesia, and axillary blocks provide effective anesthesia distal to the elbow. During brachial plexus hydrodissection for chronic pain management, the interscalene or supraclavicular approaches are typically used because these are two very common entrapment points for the brachial plexus [41]. The choice of approach depends on how proximal the cause of the neuropathic pain is. If the entrapment/neurological injuries are at the cervical root levels, interscalene brachial plexus hydrodissection is recommended. If the cause of the neuropathic pain is at the trunk or division level of the brachial plexus or if there is an excessive upward movement of the shaft of the first rib due to excessive pulling of the anterior and middle scalene, supraclavicular brachial plexus hydrodissection may provide better relief.

### 3.2. Interscalene Approach

**Figure 6:**
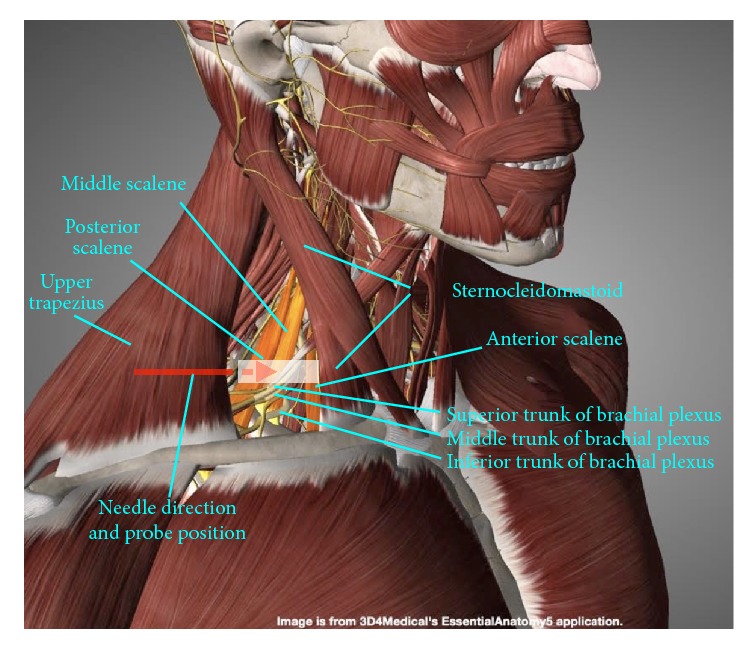
Procedure for interscalene brachial plexus hydrodissection: gross anatomy. Muscular landmarks, probe position, and needle orientation during interscalene brachial plexus hydrodissection.

*Muscular Landmarks*. The anterior, middle, and posterior scalenes are identified (Figure 6). The interscalene brachial plexus is generally formed by the C5, C6, C7, and C8 nerve roots. The needle is inserted in a direction posterior to anterior and lateral to medial, and it passes through the middle scalene to reach the interscalene brachial plexus.

**Figure 7:**
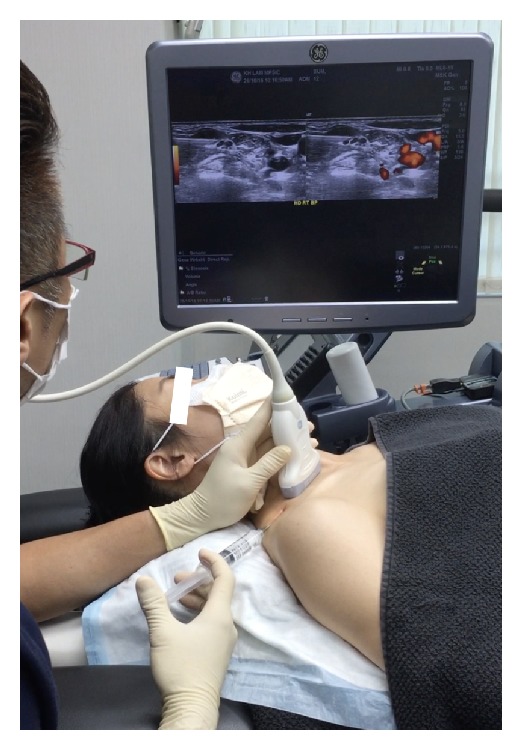
Procedure for interscalene brachial plexus hydrodissection: patient positioning. Neck support, ipsilateral shoulder elevation, and probe and needle positions for interscalene brachial plexus hydrodissection.

*Patient and Probe Positions*. The patient is supine, with a rolled towel underneath the neck for slight extension and the head is straight up or slightly rotated to the side contralateral to the point of needle entry (Figure 7). The probe is transverse to the neck.

**Figure 8:**
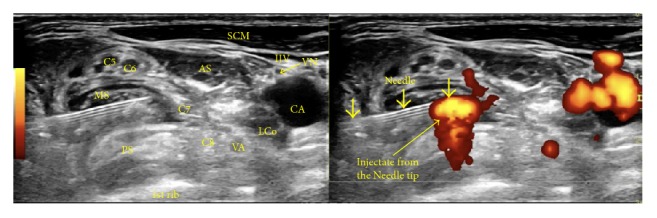
Procedure for interscalene brachial plexus hydrodissection: sonoanatomy. The image shows needle penetration through the middle scalene, with hydrodissection and fluid injection in the interscalene brachial plexus. AS: anterior scalene, CA: carotid artery, IJV: internal jugular vein, LCo: longus colli, MS: middle scalene, PS: posterior scalene, SCM: sternocleidomastoid, VA: vertebral artery, and VN: vagus nerve.

*Sonoanatomy*. Visualize the scalenes and the hypoechoic oval nerve roots of C5–C8, which are situated between the anterior and middle scalenes. The pertinent sonoanatomy is shown in Figure 8.

**Figure 9:**
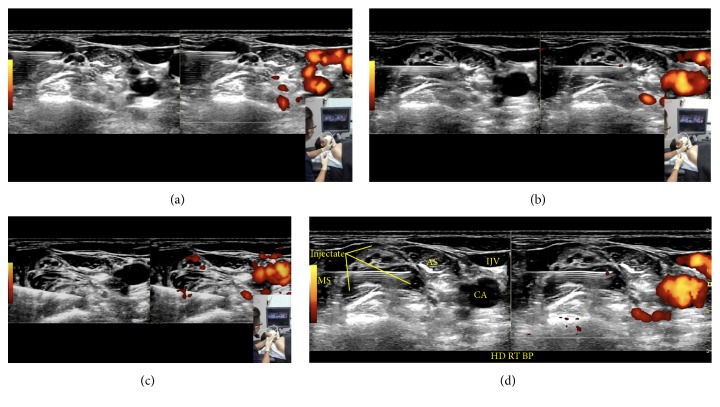
(a) Procedure for interscalene brachial plexus hydrodissection: upper trunk. Needle positioning for hydrodissection of the C5 and C6 nerve roots/upper trunk. (b) Procedure for interscalene brachial plexus hydrodissection: middle trunk. Needle positioning for hydrodissection of the C7 nerve root/middle trunk. (c) Procedure for interscalene brachial plexus hydrodissection: lower trunk. Needle positioning for hydrodissection of the C8/T1 nerve root/lower trunk. (d) Procedure for interscalene brachial plexus hydrodissection: visualization of the injectate. Visualization of the injectate during hydrodissection of the middle trunk.

*Needle Advancement/Injection (Figures 9(a)–9(c))*. An in-plane approach is used, with the needle advancing in a direction from posterior to anterior and lateral to medial (Figures 6 and 7). At the interscalene level, the cervical nerve roots start to form the superior trunk (C5/6), middle trunk (C7), and inferior trunk (C8/T1). The fascial sheath for these trunks is formed from the fascia of the surrounding scalenes. To perform interscalene brachial plexus hydrodissection, one needle entry point is typically used, and the needle should hydrodissect its way into the fascial sheath of each trunk. Once inside the fascial sheath, the injectate will surround the trunk effectively, although occasionally, hydrodissection above and below becomes necessary to separate the fascia from the trunk.  Figure 9(d) shows visualization of the injectate during hydrodissection, and video 2 shows the procedure for interscalene brachial plexus hydrodissection.

### 3.3. Supraclavicular Approach

**Figure 10:**
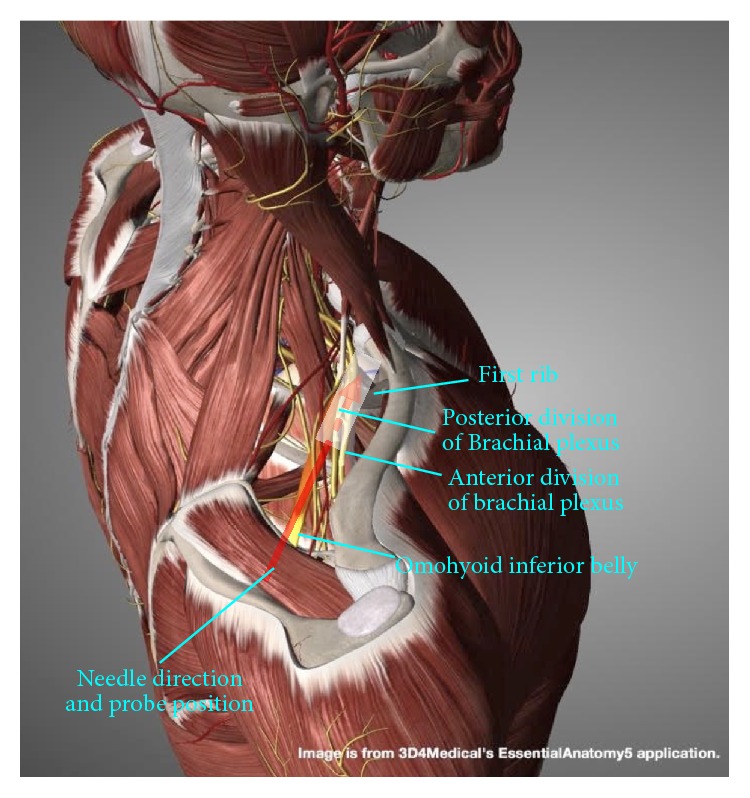
Procedure for supraclavicular brachial plexus hydrodissection: gross anatomy. Gross anatomy and needle direction for supraclavicular brachial plexus hydrodissection.

**Figure 11:**
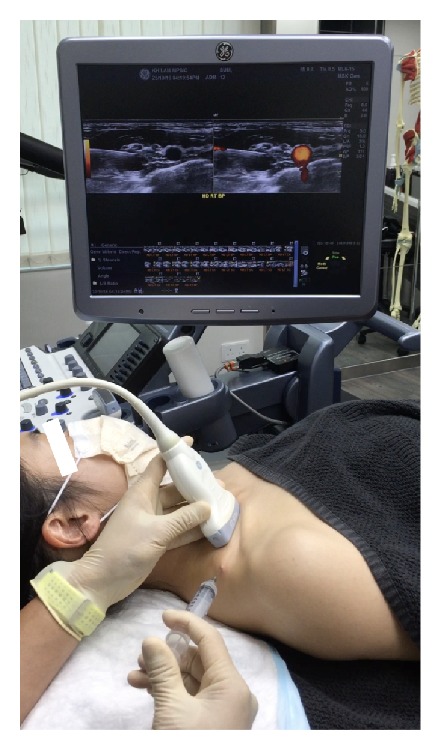
Procedure for supraclavicular brachial plexus hydrodissection: patient positioning. Neck support, ipsilateral shoulder elevation, and probe and needle positions for supraclavicular brachial plexus hydrodissection.

The anterior and middle scalenes may be traced to their insertions on the first rib, and the entire brachial plexus will gather on top of the first rib as the supraclavicular brachial plexus, lateral to the subclavian artery (Figures 10 and 11).

*Patient and Probe Positions*. The patient is supine, with a rolled towel underneath the neck for slight extension and another thin pillow or layers of towel beneath the ipsilateral shoulder for slight rotation of the entire trunk and neck to the side contralateral to the point of needle entry (Figure 11). The probe is transverse to the trunk and nearly parallel to the clavicle. An in-plane approach is used, with the needle advancing in a direction from posterior to anterior and lateral to medial.

**Figure 12:**
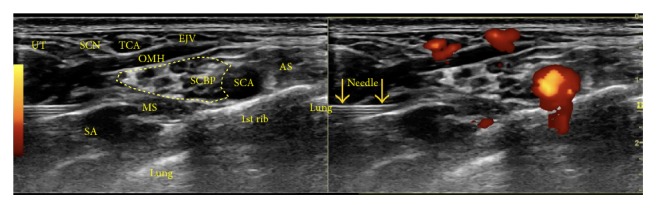
Procedure for supraclavicular brachial plexus hydrodissection: sonoanatomy. Sonographic view of the needle approaching the supraclavicular brachial plexus. AS: anterior scalene, EJV: external jugular vein, MS: middle scalene, OMH: omohyoid, SA: serratus anterior, SCA: subclavian artery, SCBP: supraclavicular brachial plexus, SCN: supraclavicular nerve, TCA: transverse cervical artery, and UT: upper trapezius.

*Sonoanatomy*. Visualize the brachial plexus gathered on top of the first rib, with the subclavian artery on the medial side (Figure 12).

**Figure 13:**
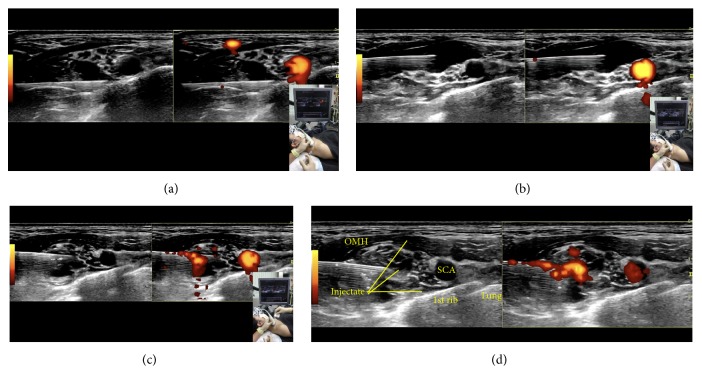
(a) Procedure for supraclavicular brachial plexus hydrodissection: first position. Needle positioning for hydrodissection of the bottom of the supraclavicular brachial plexus. (b) Procedure for supraclavicular brachial plexus hydrodissection: second position. Needle positioning for hydrodissection of the top of the supraclavicular brachial plexus. (c) Procedure for supraclavicular brachial plexus hydrodissection: third position. Needle positioning for hydrodissection of the middle of the supraclavicular brachial plexus. (d) Procedure for supraclavicular brachial plexus hydrodissection: visualization of the injectate. The image shows the anechoic injectate.

*Needle Advancement and Hydrodissection*. Visualize the brachial plexus gathered on top of the first rib, with the subclavian artery on the medial side. Figures 13(a)–13(c) show sequential needle placement for hydrodissection below, above, and between portions of the supraclavicular brachial plexus. If the patient achieves good pain relief with hydrodissection below and above the brachial plexus, a third-needle placement does not appear to be necessary.  Figure 13(d) shows the anechoic injectate during hydrodissection, and video 3 shows the procedure for supraclavicular brachial plexus hydrodissection.

#### 3.3.1. Cervical Nerve Root Hydrodissection

*Indications*. Selective cervical nerve root blocks play an important role in the conservative treatment of patients with cervical radicular pain [42]. In chronic pain management, hydrodissection of selective cervical nerve roots has been used to treat patients with postherpetic neuropathic pain involving the dermatome of specific cervical nerve roots, patients with postradiation neuritis, and patients with nerve compression from fibrosis of the neck muscles.

**Figure 14:**
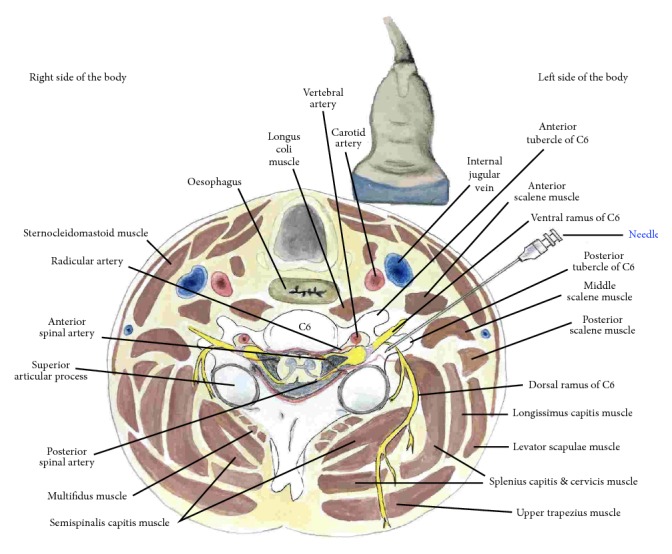
Procedure for cervical nerve root hydrodissection: cross-sectional anatomy. Cross-sectional anatomy at the C6 level.

*Bony Landmarks*. Bony landmarks include the hyperechoic anterior and posterior tubercles of the cervical vertebra, noting that C7 has no anterior tubercle and C8 has no anterior or posterior tubercle.  Figure 14 shows the cross-sectional anatomy at the C6 level for C6 root hydrodissection.

**Figure 15:**
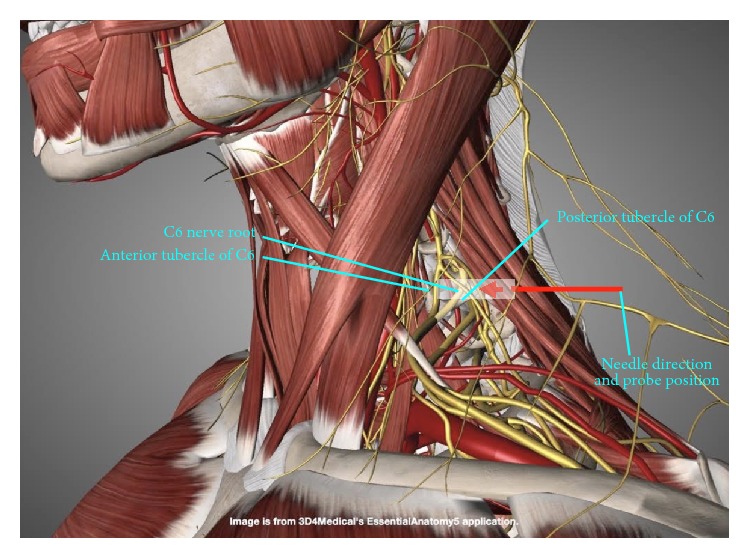
Procedure for cervical nerve root hydrodissection: gross anatomy. Gross anatomy and probe and needle directions for supraclavicular brachial plexus hydrodissection. The probe position is indicated by the white rectangle.

**Figure 16:**
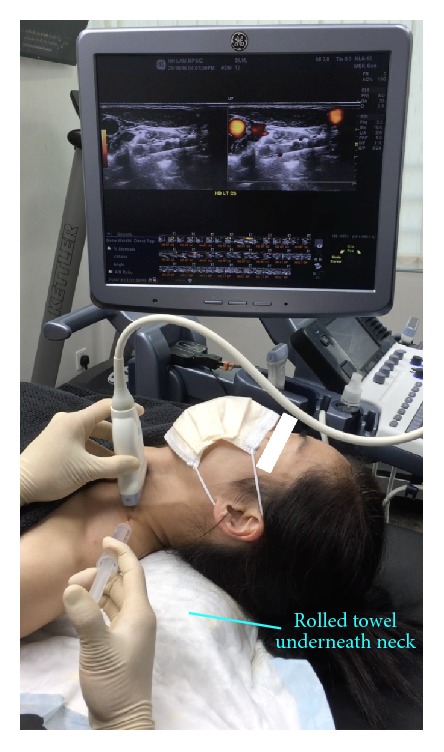
Procedure for cervical nerve root hydrodissection: patient positioning. Neck support, ipsilateral shoulder elevation, and probe and needle positions for cervical nerve root hydrodissection.

*Patient and Probe Positions*. The patient is supine, with the neck straight or slightly tilted to the contralateral side. The probe is transverse to the neck (Figures 15 and 16). An in-plane approach is used, with the needle advancing in a direction from posterior to anterior and lateral to medial.

**Figure 17:**
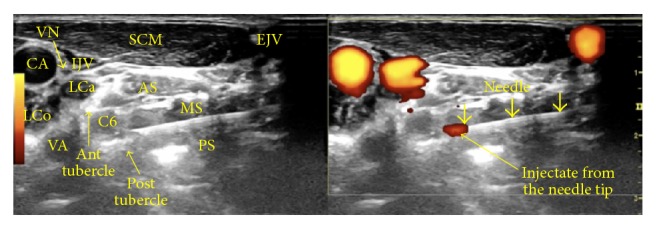
Procedure for cervical nerve root hydrodissection: sonoanatomy. Ant: anterior, Post: posterior, AS: anterior scalene, MS: middle scalene, PS: posterior scalene, CA: carotid artery, IJV: internal jugular vein, EJV: external jugular vein, VA: vertebral artery, SCM: sternocleidomastoid, VN: vagus nerve, LCa: Longus Capitis, and LCo: longus colli. The left side shows a B-mode image and the right side shows a power Doppler view.

*Sonoanatomy*.  Figure 17 is a snapshot showing the needle passing between the middle and posterior scalenes or, in some cases, only through the middle scalene. The needle tip is almost touching the posterior tubercle of the C6 transverse process.

**Figure 18:**
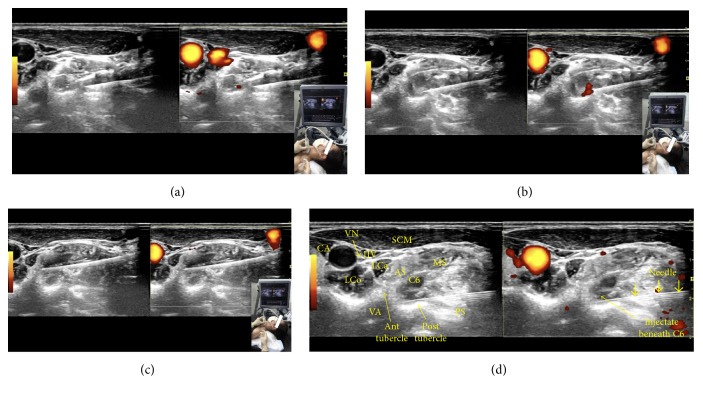
(a) Procedure for C6 nerve root hydrodissection: position one. Hydrodissection while approaching the C6 nerve root. (b) Procedure for C6 nerve root hydrodissection: position two. Needle positioning for hydrodissection dorsal to the C6 nerve root. (c) Procedure for C6 nerve root hydrodissection: position three. Needle positioning for hydrodissection ventral to the C6 nerve root. (d) Procedure for C6 nerve root hydrodissection: visualization of the injectate. The image shows the anechoic injectate after C6 nerve root hydrodissection.

*Needle Advancement and Hydrodissection*. Visualize the hypoechoic nerve roots situated between the anterior and posterior tubercles of the transverse processes of the cervical vertebrae. As illustrated in the representative image of C6 nerve root hydrodissection (Figure 18), the needle tip stops at the posterior tubercle to hydrodissect the soft tissue around the C6 nerve roots to the point where the injectate surrounds the entire nerve root. Typically, 20–30 ml of D5W is used to achieve satisfactory pain relief with fluid surrounding the nerve root. Exercise caution during C7 cervical nerve root hydrodissection, because C7 does not have an anterior tubercle. Ensure that power Doppler view is switched on to avoid mistaking the vertebral artery for the C7 nerve root.  Figure 18(d) shows the anechoic injectate after hydrodissection of the C6 nerve root, and video 4 shows the procedure for C6 nerve root hydrodissection.

#### 3.3.2. Paravertebral Hydrodissection

*General Indications*. Paravertebral block involves injection of a local anesthetic in a space immediately lateral to the point of emergence of the spinal nerves from the intervertebral foramina. This technique is increasingly being used for both intra- and postoperative analgesia and as a sole anesthetic technique for various procedures. Its popularity is mainly attributed to the ease of performance and lower complication rate when compared with techniques using catheters.

*Hydrodissection Applications*. In our experience, paravertebral hydrodissection has been observed to result in analgesia in patients who present with acute herpes zoster, prevent the development of postherpetic neuralgia, and benefit patients with established postherpetic neuralgia. This analgesic effect is consistently noted within 5 min of procedure completion and is often noted within seconds. It peaks within 30 min, maintains its peak for 2–4 h, and declines over 48 h, with a common residual effect of 10%–20% at 4 weeks.

**Figure 19:**
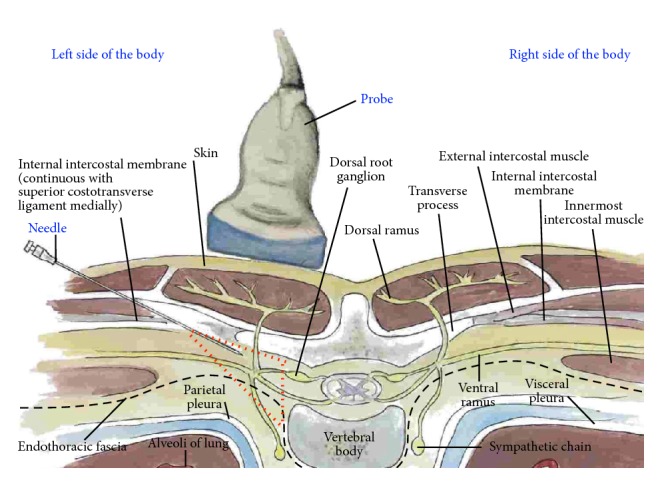
Procedure for paravertebral hydrodissection: cross-sectional anatomy. The red dashed triangle is an approximation of the paravertebral space.

*Pictorial Anatomy*. The target of this technique has been postulated to be the wedge-shaped paravertebral space whose boundaries were defined by Klein et al. [43] using a small (2.3 mm) fiber optic scope. These boundaries include the parietal pleura ventrolaterally; heads of the ribs, transverse process, and superior costotransverse ligament dorsally; and vertebra, intervertebral discs, and intervertebral foramina medially. There is a lateral extension in continuity with the intercostal space (Figure 19). A single injection into this space accesses not only the ventral and dorsal rami but also the sympathetic chain and gray rami communicantes. The advantage of using ultrasound guidance for injection into the paravertebral space has been described by Batra et al. [44].

**Figure 20:**
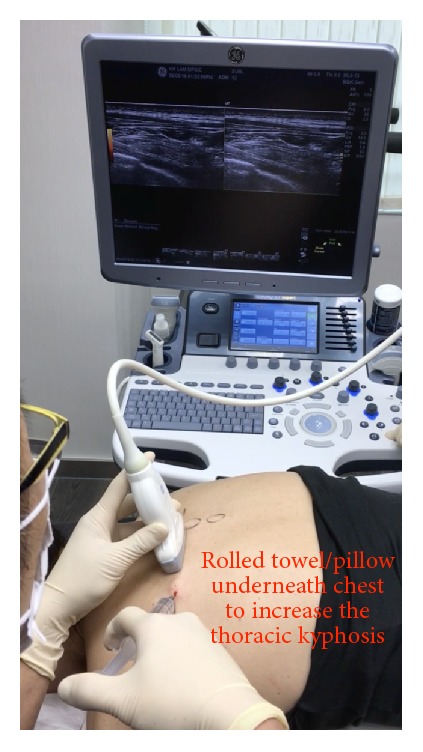
Procedure for paravertebral hydrodissection: patient positioning. Rounded back and needle and probe positions for thoracic paravertebral hydrodissection.

*Patient and Probe Positions*. The patient is prone, with a rolled towel or pillow underneath the chest to increase the degree of thoracic kyphosis (Figure 20). The probe position is transverse to the trunk, parallel to the ribs above, and below the transverse process.

**Figure 21:**
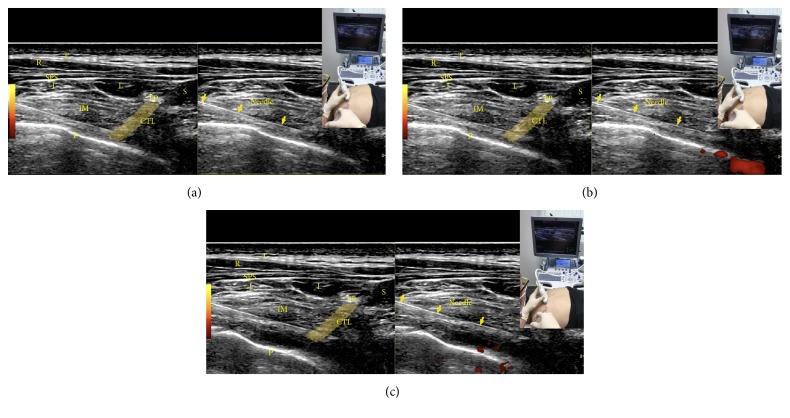
(a) Procedure for paravertebral hydrodissection: needle approaching the paravertebral space. CTL: costotransverse ligament, I: iliocostalis, IM: intercostal muscles, L: longissimus, P: pleura, R: rhomboid, S: spinalis, T: trapezius, SPS: serratus posterior superior, and TP: inferior edge of the transverse process as observed by volume averaging. The yellow band is centered over the costotransverse ligament. The yellow solid arrows show the needle. (b) Procedure for paravertebral hydrodissection: needle tip through the costotransverse ligament. Stop advancing the needle just as the tip passes the costotransverse ligament. (c) Procedure for paravertebral hydrodissection: visualization of the pleura. Pleura being pushed away by fluid.

*Pertinent Sonoanatomy*.  Figure 21(a) shows the surrounding structures when the transducer is placed immediately caudal to the costotransverse joint. Because the probe has a width and all the three-dimensional information scanned beneath the probe will be processed by the computer to be presented as a two-dimensional image on the monitor, the tip of the transverse process, which is not in the same plane of the needle and injection, will often appear as if it is in the same plane, providing additional information to confirm the costotransverse ligament position through visualization of its superomedial origin on the transverse process.

*Needle Advancement and Hydrodissection*. Hydrodissect while advancing the needle through the external and internal intercostals (Figure 21(a)). A 22-gauge needle is preferable to a 25-gauge needle, because a 22-gauge needle may provide a feeling of penetration. Stop advancing when penetration is felt or when the needle tip is observed to just pass through the costotransverse ligament (Figure 21(b)). With the needle tip beneath the lateral tip of the transverse process, further hydrodissection should be accompanied by visualization of the parietal pleura pushing away to confirm paravertebral space injection (Figure 21(c)). The fluid should then be able to access the nerve root and dorsal root ganglion, which are the targets for chronic pain control, considering the pleura forms the floor of the paravertebral space. Video 5 shows the procedure for paravertebral space hydrodissection.

Treatment of two to three levels of the paravertebral nerve roots is generally necessary for complete pain relief. To determine the thoracic spinal level by ultrasound, a paramedian sagittal view with the transducer in cross section to the transverse processes of the thoracic vertebrae and ribs is utilized to count down from the first rib or up from the twelfth rib. Video 6 explains how to use ultrasound to count the levels of the thoracic spinal nerves.

Empirically, one to two treatments are required for acute pain relief, with the second administered after rash subsidence to prevent the development of postherpetic neuralgia. In patients with established postherpetic neuralgia, pain scores will drop to 0–3/10 or to a tolerable level immediately after each injection and gradually increase thereafter, albeit with some cumulative effect. Four to six injections typically result in pain scores of 1-2/10.

## 4. Results

### 4.1. Retrospective Data Collection

**Figure 22:**
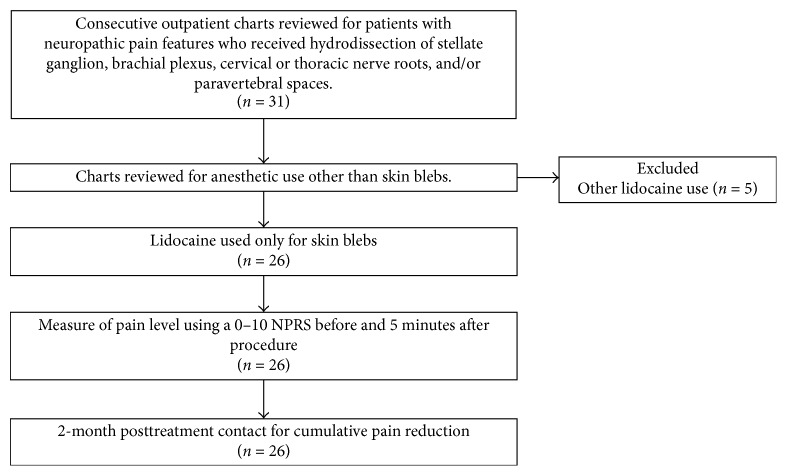
Flowchart for data collection. Retrospective data collection diagram.

Figure 22 depicts the flow chart for data selection in this retrospective study. In total, 30 consecutive patients who received D5W as the primary injectate during hydrodissection for neuropathic pain in the upper body were included. Of these, five patients requested the use of lidocaine in the injectate at some point during their treatment course because of injection discomfort. The remaining 25 patients (26 cases after considering two treatment sides in the patient with bilateral treatment) received lidocaine only for the placement of subcutaneous anesthetic blebs to numb the needle entry point. Data for 100 consecutive hydrodissection sessions performed in these 26 cases was collected by telephonic follow-up at 2 months after the last treatment session. Data capture was 100% up to the 2-month follow-up time point. All procedures were performed in Hong Kong at the office of the primary author between March 31, 2015, and December 29, 2016.

### 4.2. Demographics

Baseline demographics for the 26 cases are shown in Table 1. The sex distribution was even and the patients were middle-aged. The pain duration was 6 months or more except three with acute zoster pain (3 days, 3 days, and 1 week, resp.) and two with acute thoracic outlet symptoms (1 month each). Baseline pain was moderately severe to severe in this group. Only 1 patient rated their pain as less than 8.0.

### 4.3. Selection of Treatment Method according to the Primary Diagnosis and Area of Pain

Table 2 lists the number of cases with each primary diagnosis. Multiple diagnoses were frequent; diagnoses other than the primary diagnosis are mentioned in parentheses in the column titled “neuropathic pain areas.” The hydrodissection method was selected on the basis of the area of neuropathic pain and the other diagnoses.

### 4.4. Intrasession Effects of D5W

The consistency of postprocedural analgesia was strong. The minimum degree of pain reduction for the 100 procedures was 69%, with 35 procedures resulting in 100% pain reduction. The mean degree of pain reduction at 5 min after the last injection was administered across all 100 treatment sessions for the 26 cases was 88.1 ± 9.8%.

### 4.5. Cumulative Effects of D5W

**Figure 23:**
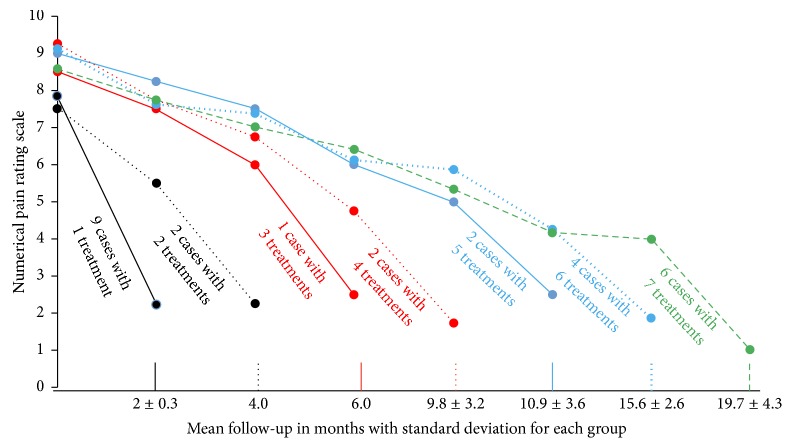
Graph showing the time course of changes in pain caused by hydrodissection with 5% dextrose water for chronic neuropathic pain. Time course of changes in 0–10 NPRS for all 26 cases (25 patients; one underwent bilateral treatment) grouped according to the number of treatment sessions. The last dot on each line represents the final pain score obtained by telephonic interview at 2 months after the last treatment session. The other dots at the beginning and along each line represent the mean pain scores for that group at each point during treatment.

From baseline to the 2-month posttreatment follow-up, a total of 3.8 ± 2.6 treatments were performed over 9.7 ± 7.8 months.  Figure 23 is a graph showing changes in pain levels over time for all 26 cases. Each line represents the changes in the mean NPRS over time for cases that received the same number of treatments. For example, the middle line shows the findings for two cases that received four treatments. When all 26 cases were combined for analysis, the mean NPRS improved from 8.3 ± 1.3 before treatment to 1.9 ± 09 after treatment, with an improvement of 6.4 ± 1.7 points. The degree of pain improvement exceeded 50% in all cases and 75% in 50% (13/26).

### 4.6. Effects of D5W Hydrodissection on Patients with Acute Pain

Of the 26 cases, 21 had pain for more than 6 months and five had pain for less than 2 months. Cases of acute pain received only one treatment, and the degree of pain reduction 5 minutes after the last injection was 97.0%  ±  6.9% in cases of acute pain and 87.7%  ±  9.8% in cases of chronic pain (*p* = .04). The mean improvement in NPRS at the 2-month posttreatment follow-up was 5.8 ± 1.9 points for cases of acute pain (8.0 ± 1.3 to 2.2 ± 0.7) and 6.5 ± 1.7 points for cases of chronic pain (*p* = .385).

## 5. Discussion

In the present study, we proposed and illustrated potentially reproducible methods of hydrodissection of the stellate ganglion, brachial plexus, cervical nerve roots, and paravertebral spaces. Salient methods common to all approaches included the following.

(1) One method is use of a skin bleb to eliminate pain at the point of needle entry.

(2) Another method is use of a 22- to 25-gauge needle, with minimization of the probe to needle angle and an emphasis on a needle in-plane approach to maximize needle visibility.

(3) Another one is constant hydrodissection, with marked reduction of any discomfort through dissection of soft tissue in front of the needle to lead the needle, rather than splitting of the soft tissue by the needle itself, as well as further improvement of needle tip visualization.

(4) Another method is an emphasis on D5W use without lidocaine to eliminate any possibility of intravascular anesthetic injection during the hydrodissection procedure.

If an anesthetic is preferred by a patient because of discomfort, a mixture of D5W and a low dose anesthetic, for example, 0.1%–0.2% lidocaine, can be injected along the needle track before the target area is reached, followed by a switch to D5W alone so that a higher volume can be instilled for the bulk of the hydrodissection procedure for the target nerve structures. However, although small doses of lidocaine may help in increasing patient comfort, the physician is advised to limit lidocaine application during stellate ganglion hydrodissection because of potential changes in vagal modulation and baroreceptor sensitivity [45] and during deep cervical plexus hydrodissection because of potential effects on the recurrent laryngeal nerve, particularly if the patient has an unrecognized baseline dysfunction of the contralateral recurrent laryngeal nerve or if bilateral hydrodissection is required [46].

(5) One of them is slow needle advancement, which allows the injectate to dissect the tissue layer by layer until the nerve/plexus is reached, with emphasis on precise visualization of the needle tip when the needle is approaching nerves and blood vessels.

(6) Another one is fluid delivery above and below the nerve for more complete hydrodissection.

This should preferably start just below the nerve, because if there is any air in the injectate, the acoustic shadow of the air will not block the view.

This retrospective data collection provides preliminary data which supports a consistent analgesic effect of D5W across a variety of neuropathic pain conditions and a cumulative benefit of repeated D5W hydrodissection. An important observation was that the onset speed of analgesia was fast enough that pain relief could be used to determine whether the procedure was sufficiently complete. The effect of D5W injection in patients with chronic neuropathic pain in the present study was similar, in both the speed of onset and magnitude of analgesic effect, to that in a recent randomized controlled trial of D5W versus saline injection in the epidural space of patients with chronic low back pain with various etiologies [17]. In that study, saline exhibited no analgesic effects. Moreover, the cumulative effect of repeated D5W hydrodissection was consistent with that in a prospective trial of epidural D5W injection [18]. However, the follow-up period in the present study was only 2 months after treatment completion. A prospective study with a long-term follow-up period and preferably including a control group is necessary to confirm our findings. The study should be of sufficient size to effectively compare treatment outcomes between patients with acute pain and those with chronic pain.

The mechanism of action of dextrose-induced analgesia is not clear, although research supports several hypotheses. First, dextrose may act at the level of pain receptors. Chronic neuropathic pain is associated with persistent upregulation of the transient receptor potential vanilloid receptor-1 (TRPV1) ion channel [47], which is upregulated by capsaicin. Mannitol (an analog of dextrose) application to the lip reduced the burning pain associated with capsaicin application in a lip pain model [48], and in our experience, dextrose has a similar effect. A class effect of sugars to indirectly reduce the effects of TRPV1 receptor activation is proposed, because neither dextrose nor mannitol has a known binding point to the TRPV1 receptor [49].

Second, extracellular dextrose elevation may hyperpolarize normoglycemic C fibers, lowering their firing rate. Dextrose elevation to 0.5% (from the normal blood level of 0.1) in the intestinal lumen rapidly results in hyperpolarization of enterocytic cell membranes to facilitate transport across the cell membrane by sodium glucose cotransporter (SGLT1) [50]. In peripheral nerves, the primary glucose transport is via glucose transporter one, not SGLT1 [51]. However, SGLT1 is still present on neuronal cell membranes [51]. The effect of a 50-fold increase in extracellular dextrose (D5W) on SGLT activity in normoglycemic C fibers has not been directly studied. However, recent reports on the coadministration of D5W to decrease the pain from infusion of chemotherapeutic agents [13, 14] or microspheres [15] point to a potential analgesic effect in normoglycemic subjects, although the mechanism remains to be confirmed.

Third, dextrose may reverse a proposed energy-deficient state of neuropathic nerves. A decrease in blood dextrose of only 25% (1.5 mM) from the normal fasting range in rats is reported to initiate histopathological changes in the peripheral nervous system [52] long before blood levels that will initiate brain damage in the rat [53] or brain dysfunction in humans [54] are reached. We propose that pain is an alarm signal produced by nociceptive C fibers which begins promptly upon development of intraneural hypoglycemia, prior to onset of histopathologic changes in the C fiber. Maclver and Tanelian [55] studied action potential changes in response to hypoglycemia in C fibers of the New Zealand White rabbit cornea in vitro and noted an increase in the C fiber discharge frequency of 653%  ±  28% relative to that in a normoglycemic control within 15 min of hypoglycemia onset, followed by rapid return to normal firing levels after the administration of dextrose.

**Figure 24:**
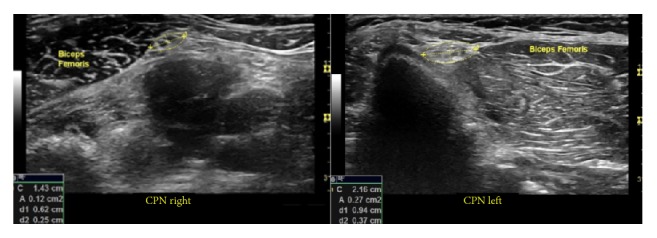
Illustration of a swollen nerve. The left image shows the left common fibular (peroneal) nerve at the level of the biceps femoris (knee). The larger right common fibular nerve is seen on the right side. The area was calculated using ultrasound measurement tools.

The theoretical basis for the clinical benefits of hydrodissection is compelling. Bennett and Wie developed an animal model of neuropathic pain caused by chronic constriction injury, which is widely utilized and involves the application of a ligature that is barely snug around the sciatic nerve [56]. Specific recommendations for the induction of neuropathic pain are as follows: use a high-power objective lens to observe the flow of red blood cells in the epineural vasculature and tie the ligature just tight enough for the flow to slow down without stopping or tighten the ligature so that it will slide along the nerve, but not smoothly. The result is the development of a neural swelling, typically within 24 h, on both sides of the ligature, accompanied by classic findings of neuropathic pain such as hyperalgesia, allodynia, and, frequently, dysesthesia [56]. A similar swelling of nerves, accompanied by a graded compromise in the vascular nerve supply, is notable on high-resolution ultrasound examinations and is strongly associated with nerve compression, such as that occurring in carpal tunnel syndrome [57]. Ultrasound examination of peripheral nerves in the presence of neuropathic pain commonly demonstrates an increase in individual nerve fascicle size, an increase in neural volume, or, typically, both [21]. An example is shown in Figure 24, which depicts the smaller left and larger right common fibular (peroneal) nerve at the level of the knee in the same patient (symptomatic on the right side). Studies with large or formal data collection procedures showing changes in fascicular swelling in response to D5W injection have not yet been reported.

Future studies, particularly prospective studies are necessary to evaluate the frequency of intraneural edema in various neuropathic pain syndromes, long-term efficacy of hydrodissection in comparison to that of standard-volume anesthetic blocks, and use of injectates other than D5W, such as platelet-rich plasma, which may have a favorable effect on dysfunctional nerves by itself [58]. In addition, because the amount of compression necessary to create a chronic constriction effect appears to be minimal [56], it is important to consider all possible points of constriction on these predominantly small-fiber sympathetic nerves between their peripheral origin and central process entry into the neural foramina, without restriction to the classic entrapment locations.

The potential importance of the analgesic effect of dextrose in the absence of anesthetic should not be overlooked in clinical applications and research. Compared with a nerve block with anesthetic, injection of dextrose for diagnostic purposes may provide a more precise method for identifying which branch or portion of a peripheral nerve is the nociceptive source within the nerve tree because it does not depolarize the nerve [57].

## 6. Conclusions

In conclusion, we described and illustrated potentially reproducible methods of hydrodissection of the stellate ganglion, brachial plexus, cervical nerve roots, and paravertebral spaces, provided data supporting a consistent analgesic effect of D5W used as the primary injectate, and suggested a potentially sustainable clinical benefit in patients with chronic upper back/thoracic pain of neuropathic origin. The mechanism of analgesia may be related to an indirect (allosteric) effect on the TRPV1 cation channel, hyperpolarization of normoglycemic C fibers, correction of local neural hypoglycemia, or undiscovered, probably multiple, mechanisms. The well-developed chronic constriction injury model, which results in neuropathic pain and neural swelling, is the primary rationale behind hydrodissection to release the nerve from suspected local neural compression, particularly those nerves with fascicular swelling or an increase in the overall volume. The frequency of neural edema and the long-term efficacy for nerve hydrodissection in patients with neuropathic pain, as opposed to those for low-volume anesthetic nerve blocks, are important foci for future research on neuropathic pain conditions.

## Supplementary Material

Video 1 shows the actual procedure for stellate ganglion hydrodissection using D5W.
Video 2 shows the actual procedure for interscalene brachial plexus hydrodissection using D5W.
Video 3 shows the actual procedure for supraclavicular brachial plexus hydrodissection using D5W.
Video 4 shows the actual procedure for C6 nerve root hydrodissection using D5W.
Video 5 shows the actual procedure for paravertebral space hydrodissection using D5W.
Video 6 explains how to use ultrasound to count the levels of the thoracic spinal nerves.

## Figures and Tables

**Table 1:** Baseline demographics. These 26 cases (25 patients, with one receiving treatment bilaterally) represent 100 consecutive hydrodissection procedures.

Baseline demographics (*n* = 26)	
Female, *n* (% of procedures)	13 (50%)
Age years, mean (SD)	51 ± 14.7
Pain duration months, mean (SD)	16 ± 12.2
NRS pain prior to 1st injection, mean (SD)	8.3 ± 1.3

**Table 2:** Diagnoses, neuropathic pain regions, and hydrodissection performed for 26 cases in 25 patients.

*n* ^a^	Primary diagnosis	Neuropathic pain areas (other diagnoses)	Regional hydrodissection^a^				
			PV^b^	SG^c^	SCBP^d^	ISBP^e^	CR^f^
3	Cervical root compression	Neck and arm			2	3	3
2	Cervical root compression	Neck					2
1	Postherpetic neuralgia	Neck					1
3	Postherpetic neuralgia	Chest	3				
3	Acute herpes zoster	Neck					1
3	Acute herpes zoster	Chest	2				
2	Thoracic outlet syndrome	Arm			2		
1	Thoracic outlet syndrome	Arm (panic attacks)^g^		1	1		
1	Thoracic outlet syndrome	Arm (triple crush syndrome)			1		
1	Thoracic outlet syndrome	Neck and arm (double crush syndrome) (cervical radiculopathy)			1		1
1	Neuropathic pain in the head and neck region	Head and neck (PTSD)^h^		1			
1	Neuropathic pain in the head and neck region	Head and neck (PTSD) (panic attacks)		1			
1	Cervicogenic headache	Head and neck (PTSD) (panic attacks)		1			
1	Stretch injury to the brachial plexus	Arm and hand (PTSD) (CRPS)		1	1		
1	Chronic regional pain syndrome (CRPS)	Arm and hand (double crush syndrome)		1	1		
1	Neuropathic pain in the thoracic region	Thorax (after rib fracture)	1				
1	Neuropathic pain in the thoracic region	Thorax (after electric shock)	1				
1	Neuropathic pain in the arm	Neck, arm, and hand			1	1	1
1	Cervical sprain	Neck and arm		1	1		

^a^ *n* = number of cases with the same combination of diagnoses. Not all cases with the same diagnoses underwent hydrodissection in the same region. The number of cases receiving a given hydrodissection type is listed in each column under the main column titled “Regional hydrodissection.” ^b^PV = paravertebral hydrodissection, typically performed for neuropathic pain in the thoracic region. ^c^SG = stellate ganglion hydrodissection, typically performed for CRPS and other chronic neuropathic pain conditions involving the head and neck region. ^d^SCBP = supraclavicular brachial plexus hydrodissection, typically performed for CRPS, other chronic neuropathic pain conditions involving the ipsilateral upper limb, and double crush syndrome involving the ipsilateral upper limb. ^e^ISCP = interscalene brachial plexus hydrodissection, typically performed for CRPS and other chronic neuropathic pain conditions involving the ipsilateral upper limb, particularly areas closer to the nerve roots. ^f^CR = cervical root hydrodissection, typically performed for nerve root compression of any kind and neuropathic pain involving specific nerve roots. ^g^Although panic attacks were not a primary diagnosis, they were recorded because of their frequent exacerbation by chronic pain and treatment by stellate ganglion hydrodissection. ^h^PTSD = posttraumatic stress disorder, which was not the primary diagnosis but was recorded because of its frequent association with chronic pain and treatment by stellate ganglion hydrodissection.

## References

[1] Wadhwa A., Kandadai S. K., Tongpresert S., Obal D., Gebhard R. E. (2011). Ultrasound guidance for deep peripheral nerve blocks: a brief review. *Anesthesiology Research and Practice*.

[2] Dach F., Éckeli Á. L., Ferreira K. D. S., Speciali J. G. (2015). Nerve block for the treatment of headaches and cranial neuralgias—a practical approach. *Headache*.

[3] Clendenen S., Greengrass R., Whalen J., O’Connor M. I. (2015). Infrapatellar saphenous neuralgia after TKA can be improved with ultrasound-guided local treatments. *Clinical Orthopaedics and Related Research*.

[4] Cass S. P. (2016). Ultrasound-guided nerve hydrodissection: what is it? a review of the literature. *Current Sports Medicine Reports*.

[5] Neal J. M., Barrington M. J., Brull R. (2015). The second ASRA practice advisory on neurologic complications associated with regional anesthesia and pain medicine: executive summary 2015. *Regional Anesthesia and Pain Medicine*.

[6] Hara K., Sakura S., Yokokawa N., Tadenuma S. (2012). Incidence and effects of unintentional intraneural injection during ultrasound-guided subgluteal sciatic nerve block. *Regional Anesthesia and Pain Medicine*.

[7] Liu S. S., Yadeau J. T., Shaw P. M., Wilfred S., Shetty T., Gordon M. (2011). Incidence of unintentional intraneural injection and postoperative neurological complications with ultrasound-guided interscalene and supraclavicular nerve blocks. *Anaesthesia*.

[8] Lyftogt J. (2008). Pain conundrums: which hypothesis? Central nervous system sensitization versus peripheral nervous system autonomy. *Australasian Musculoskeletal Medicine*.

[9] Lyftogt J. (2007). Subcutaneous prolotherapy for Achilles tendinopathy. *Australasian Musculoskeletal Medicine*.

[10] Lyftogt J. (2007). Subcutaneous prolotherapy treatment of refractory knee, shoulder and lateral elbow pain. *Australasian Musculoskeletal Medicine*.

[11] Lyftogt J. (2008). Prolotherapy for recalcitrant lumbago. *Australasian Musculoskeletal Medicine*.

[12] Yelland M. J., Sweeting K. R., Lyftogt J. A., Ng S. K., Scuffham P. A., Evans K. A. (2011). Prolotherapy injections and eccentric loading exercises for painful Achilles tendinosis: a randomised trial. *British Journal of Sports Medicine*.

[13] Hosokawa A., Nakashima T., Ogawa Y., Kozawa K., Kiba T. (2013). Coadministration of 5% glucose solution relieves vascular pain in the patients administered gemcitabine immediately. *Journal of Oncology Pharmacy Practice*.

[14] Nakashima T., Ogawa Y., Kimura A. (2012). Coadministration of 5% glucose solution has a decrease in bendamustine-related vascular pain grade. *Journal of Oncology Pharmacy Practice*.

[15] Paprottka K. J., Lehner S., Fendler W. P. (2016). Reduced periprocedural analgesia after replacement of water for injection with glucose 5% solution as the infusion medium for 90Y-Resin microspheres. *Journal of Nuclear Medicine*.

[16] Dufour E., Donat N., Jaziri S. (2012). Ultrasound-guided perineural circumferential median nerve block with and without prior dextrose 5% hydrodissection: a prospective randomized double-blinded noninferiority trial. *Anesthesia and Analgesia*.

[17] Maniquis-Smigel L., Reeves K. D., Rosen H. J. (2017). Short term analgesic effects of 5% dextrose epidural injections for chronic low back pain: a randomized controlled trial. *Anesthesiology and Pain Medicine*.

[18] Maniquis Smigel L., Reeves K. D., Lyftogt J., Cheng A. L., Rabago D. (2016). Caudal epidural injections with 5% dextrose for chronic low back pain with accompanying buttock or leg pain: results of a consecutive participant open-label trial with long-term follow-up. *Archives of Physical Medicine and Rehabilitation*.

[19] Jensen T. S., Baron R., Haanpää M. (2011). A new definition of neuropathic pain. *Pain*.

[20] Treede R.-D., Jensen T. S., Campbell J. N. (2008). Neuropathic pain: redefinition and a grading system for clinical and research purposes. *Neurology*.

[21] Ebadi H., Siddiqui H., Ebadi S., Ngo M., Breiner A., Bril V. (2015). Peripheral nerve ultrasound in small fiber polyneuropathy. *Ultrasound in Medicine and Biology*.

[22] Ackerman III W. E., Zhang J.-M. (2006). Efficacy of stellate ganglion blockade for the management of type 1 complex regional pain syndrome. *Southern Medical Journal*.

[23] Yucel I., Demiraran Y., Ozturan K., Degirmenci E. (2009). Complex regional pain syndrome type I: efficacy of stellate ganglion blockade. *Journal of Orthopaedics and Traumatology*.

[24] Sinofsky A., Sharma T., Wright T. (2016). Stellate ganglion block for debilitating photophobia secondary to trigeminal, postherpetic neuralgia. *Pain Practice*.

[25] Makharita M. Y., Amr Y. M., El-Bayoumy Y. (2012). Effect of early stellate ganglion blockade for facial pain from acute Herpes Zoster and Incidence of postherpetic neuralgia. *Pain Physician*.

[26] Melis M., Zawawi K., Al-Badawi E., Lobo S. L., Mehta N. (2002). Complex regional pain syndrome in the head and neck: A review of the literature. *Journal of Orofacial Pain*.

[27] Arden R. L., Bahu S. J., Zuazu M. A., Berguer R. (1998). Reflex sympathetic dystrophy of the face: current treatment recommendations. *Laryngoscope*.

[28] Mulvaney S. W., Lynch J. H., Kotwal R. S. (2015). Clinical guidelines for stellate ganglion block to treat anxiety associated with posttraumatic stress disorder. *Journal of Special Operations Medicine*.

[29] Lynch J. H., Mulvaney S. W., Kim E. H., de Leeuw J. B., Schroeder M. J., Kane S. F. (2016). Effect of stellate ganglion block on specific symptom clusters for treatment of post-traumatic stress disorder. *Military Medicine*.

[30] Summers M. R., Nevin R. L. (2017). Stellate ganglion block in the treatment of post-traumatic stress disorder: a review of historical and recent literature. *Pain Practice*.

[31] Mulvaney S. W., Lynch J. H., Hickey M. J. (2014). Stellate ganglion block used to treat symptoms associated with combat-related post-traumatic stress disorder: a case series of 166 patients. *Military Medicine*.

[32] Ghai A., Kaushik T., Kundu Z. S., Wadhera S., Wadhera R. (2016). Evaluation of new approach to ultrasound guided stellate ganglion block. *Saudi Journal of Anaesthesia*.

[33] Ghai A., Kaushik T., Wadhera R., Wadhera S. (2016). Stellate ganglion blockade-techniques and modalities. *Acta Anaesthesiologica Belgica*.

[34] Gofeld M., Bhatia A., Abbas S., Ganapathy S., Johnson M. (2009). Development and validation of a new technique for ultrasound-guided stellate ganglion block. *Regional Anesthesia and Pain Medicine*.

[35] Tran Q. H., Clemente A., Doan J., Finlayson R. J. (2007). Brachial plexus blocks: a review of approaches and techniques. *Canadian Journal of Anesthesia*.

[36] Brachial Plexus Injury (BPI) at http://www.hopkinsmedicine.org/neurology_neurosurgery/centers_clinics/peripheral_nerve_surgery/conditions/brachial_plexus_injury_bpi.html

[37] Fallatah S. (2014). Successful management of complex regional pain syndrome type 1 using single injection interscalene brachial plexus block. *Saudi Journal of Anaesthesia*.

[38] Golovchinsky V. (2000). *Double-Crush Syndrome*.

[39] Vermeylen K., Engelen S., Sermeus L., Soetens F., Van De Velde M. (2012). Supraclavicular brachial plexus blocks: review and current practice. *Acta Anaesthesiologica Belgica*.

[40] Mian A., Chaudhry I., Huang R., Rizk E., Tubbs R. S., Loukas M. (2014). Brachial plexus anesthesia: a review of the relevant anatomy, complications, and anatomical variations. *Clinical Anatomy*.

[41] Pratt N. (2005). Anatomy of nerve entrapment sites in the upper quarter. *Journal of Hand Therapy*.

[42] Van Zundert J., Huntoon M., Patijn J., Lataster A., Mekhail N., Van Kleef M. (2010). Cervical radicular pain. *Pain Practice*.

[43] Klein S. M., Nielsen K. C., Ahmed N., Buckenmaier III C. C., Steele S. M. (2004). In situ images of the thoracic paravertebral space. *Regional Anesthesia and Pain Medicine*.

[44] Batra R. K., Krishnan K., Agarwal A. (2011). Paravertebral block. *Journal of Anaesthesiology Clinical Pharmacology*.

[45] Song J.-G., Hwang G.-S., Eun H. L. (2009). Effects of bilateral stellate ganglion block on autonomic cardiovascular regulation. *Circulation Journal*.

[46] Weiss A., Isselhorst C., Gahlen J. (2005). Acute respiratory failure after deep cervical plexus block for carotid endarterectomy as a result of bilateral recurrent laryngeal nerve paralysis. *Acta Anaesthesiologica Scandinavica*.

[47] Malek N., Pajak A., Kolosowska N., Kucharczyk M., Starowicz K. (2015). The importance of TRPV1-sensitisation factors for the development of neuropathic pain. *Molecular and Cellular Neuroscience*.

[48] Bertrand H., Kyriazis M., Reeves K. D., Lyftogt J., Rabago D. (2015). Topical mannitol reduces capsaicin-induced pain: results of a pilot-level, double-blind, randomized controlled trial. *PM and R*.

[49] Cui M., Gosu V., Basith S., Hong S., Choi S. (2016). Polymodal transient receptor potential vanilloid type 1 nocisensor: structure, modulators, and therapeutic applications. *Advances in Protein Chemistry and Structural Biology*.

[50] Chen L., Tuo B., Dong H. (2016). Regulation of intestinal glucose absorption by ion channels and transporters. *Nutrients*.

[51] Patching S. G. (2017). Glucose transporters at the blood-brain barrier: function, regulation and gateways for drug delivery. *Molecular Neurobiology*.

[52] Jensen V. F. H., Mølck A.-M., Bøgh I. B., Lykkesfeldt J. (2014). Effect of insulin-induced hypoglycaemia on the peripheral nervous system: focus on adaptive mechanisms, pathogenesis and histopathological changes. *Journal of Neuroendocrinology*.

[53] Jensen V. F. H., Bøgh I. B., Lykkesfeldt J. (2014). Effect of insulin-induced hypoglycaemia on the central nervous system: evidence from experimental studies. *Journal of Neuroendocrinology*.

[54] Pramming S., Thorsteinsson B., Stigsby B., Binder C. (1988). Glycaemic threshold for changes in electroencephalograms during hypoglycaemia in patients with insulin dependent diabetes. *British Medical Journal*.

[55] Maclver M. B., Tanelian D. L. (1992). Activation of C fibers by metabolic perturbations associated with tourniquet ischemia. *Anesthesiology*.

[56] Bennett G. J., Chung J. M., Honore M., Seltzer Z. (2003). Models of neuropathic pain in the rat. *Current Protocols in Neuroscience*.

[57] Dejaco C., Stradner M., Zauner D. (2013). Ultrasound for diagnosis of carpal tunnel syndrome: comparison of different methods to determine median nerve volume and value of power Doppler sonography. *Annals of the Rheumatic Diseases*.

[58] Anjayani S., Wirohadidjojo Y. W., Adam A. M., Suwandi D., Seweng A., Amiruddin M. D. (2014). Sensory improvement of leprosy peripheral neuropathy in patients treated with perineural injection of platelet-rich plasma. *International Journal of Dermatology*.

